# siRNA capsulated brain-targeted nanoparticles specifically knock down OATP2B1 in mice: a mechanism for acute morphine tolerance suppression

**DOI:** 10.1038/srep33338

**Published:** 2016-09-15

**Authors:** Zi-Zhao Yang, Li Li, Lu Wang, Ming-Cheng Xu, Sai An, Chen Jiang, Jing-Kai Gu, Zai-Jie Jim Wang, Lu-Shan Yu, Su Zeng

**Affiliations:** 1 Institute of Drug Metabolism and Pharmaceutical Analysis, Zhejiang Province Key Laboratory of Anti-Cancer Drug Research, College of Pharmaceutical Sciences, Zhejiang University, Hangzhou, 310058, China; 2 Zhejiang Provincial Key Laboratory of Geriatrics & Geriatrics Institute of Zhejiang Province, Zhejiang Hospital, 12 Lingyin Road, Hangzhou, Zhejiang Province 310013, China; 3 Key Laboratory of Smart Drug Delivery, Ministry of Education, Department of Pharmaceutics, School of Pharmacy, Fudan University, 826 Zhangheng Road, Shanghai 201203, China; State Key Laboratory of Medical Neurobiology, Fudan University, Shanghai 200032, China; 4 School of Life Sciences, Jilin Univeristy, Changchun, 130012, China; 5 Department of Biopharmaceutical Sciences, University of Illinois, 833 S. Wood Street, Chicago, IL 60612, USA

## Abstract

Regulating main brain-uptake transporter of morphine may restrict its tolerance generation, then modify its antinociception. In this study, more than 2 fold higher intracellular uptake concentrations for morphine and morphine-6-glucuronide (M6G) were observed in stable expression cells, HEK293-hOATP2B1 than HEK293-MOCK. Specifically, the *K*_*m*_ value of morphine to OATP2B1 (57.58 ± 8.90 μM) is 1.4-time more than that of M6G (80.31 ± 21.75 μM); Cyclosporine A (CsA), an inhibitor of OATP2B1, can inhibit their intracellular accumulations with IC_50_ = 3.90 ± 0.50 μM for morphine and IC_50_ = 6.04 ± 0.86 μM for M6G, respectively. To further investigate the role of OATP2B1 in morphine brain transport and tolerance, the novel nanoparticles of DGL-PEG/dermorphin capsulated siRNA (OATP2B1) were applied to deliver siRNA into mouse brain. Along with OATP2B1 depressed, a main reduction was found for each of morphine or M6G in cerebrums or epencephalons of acute morphine tolerance mice. Furthermore, calcium/calmodulin-dependent protein kinase IIα (CaMKIIα) in mouse prefrontal cortex (mPFC) underwent dephosphorylation at Thr286. In conclusion, OATP2B1 downregulation in mouse brain can suppress tolerance *via* blocking morphine and M6G brain transport. These findings might help to improve the pharmacological effects of morphine.

Tolerance is one of significant side-effects in morphine-induced antinociception^1^,^2^. Some proteins can be regulated in tolerance. For example, μ-opioid receptor (MOR) will be internalized, desensitized and auto-phosphorylated^3^,^4^; calcium outflow will activate many targets in molecular pathways, such as cyclic adenosine monophosphate (cAMP), p38 or extracellular signal-regulated kinase (ERK), then block the calcium/calmodulin-dependent protein kinase IIα (CaMKIIα) which undergoes phosphorylation at Thr286^5^,^6^. The extent of morphine tolerance can be reflected by one or more protein kinases changes or phosphatases^7^.

Morphine can be converted mainly into two metabolites, morphine-3-glucuronide (M3G) and morphine-6-glucuronide (M6G) by uridine diphosphate glucuronosyltransferase 2B7 (UGT2B7) in human^8^. Compared to morphine and M3G, M6G is much easier to induce tolerance. One report referred that daily administration of equipotent doses of M6G and morphine induced similar declines in antinociception over 9 days of treatment. To recover the initial response to identical pain, a higher dose of M6G than morphine is required in tolerant mouse^9^. In contrast, M3G may prevent tolerance to M6G^10^.

To repress morphine tolerance, one potential way is to reduce the accumulations of morphine and its metabolites in the brain by regulating their uptake or efflux. As we have known, p-glycoprotein (P-gp) can mediate morphine brain efflux^11^. P-gp induction would reduce morphine’s pharmacologic activity by increasing morphine brain efflux in rats and play main role in suppression of morphine tolerance^12^. Some researches referred both of M3G and M6G can be excreted by multidrug resistance protein 2 (MRP2, ABCC2) and multidrug resistance protein 3 (MRP3, ABCC3)^13^. There was evidence to show that MRP3 (−/−) mice lose the ability to efflux M3G from the liver into the bloodstream^14^. However, MRP2 and MRP3 barely express in the animal brains^13^,^15^. So far, a few reports described that drug transporters in brain can mediate morphine and its metabolites uptake. Recent research manifested digoxin dependent uptake transporters such as organic anion transporting polypeptides (OATPs) may be involved in morphine and its metabolites brain transport^16^,^17^. Accordingly, OATP2B1 as one of significant subtype of OATP which located in human chromosome 11 was come into our eyes^18^. It mainly expresses in the liver, gastrointestinal tract, brain and pancreas^19^,^20^,^21^ and can transfer statins, such as atorvastatin, fluvastatin, cerivastatin into the bile or blood from internal environment^22^,^23^,^24^,^25^,^26^. Since OATP2B1 is located in the endothelial cells of human brain capillaries^22^, it may potentially mediate morphine brain transport and correlate to morphine tolerance.

For understanding the functions of OATP2B1 and the relations between OATP2B1 and morphine tolerance, we expect to specifically inhibit it in mouse brain. However, it is a challenge to deliver siRNA crossing brain blood barrier (BBB) *via* non-viral system. Shyam *et al*. used a linear polyethyleneimine (LPEI)-g-polyethylene glycol (PEG) copolymer micellar nanoparticle system to capsulate siRNA and effectively knock down BACE1 in heterogeneous tissues of mice^27^. They found the nanoparticles decorated PEG can improve its brain-targeted capacity. In our study, the novel nanoparticles (NPs) were applied to capsulate and deliver siRNA specifically into mouse brain. The NPs were composed of dendrigraft poly-l-lysine (DGL), PEG and dermorphin. DGL and PEG were being as nanocarrier materials. Dermorphin is the identified aptamer, nearly 90 percent free styles of which can recognize MOR in brain parenchyma and obstructed by brain capillary endothelial cells^28^. In previously research, such formulated nanoparticle has been applied to delivery shRNA to knock down Ask1 in mice brains^29^. Compared to other non-viral nanocarriers, it owns some advantages such as injection safety, stability in circulation, low toxicity and active targeting capacity^29^. As a consequence, we selected it as brain-targeted carrier in this study to knock down OATP2B1 in mouse brain.

## Results

### OATP2B1 mediated intracellular uptake of morphine and its main metabolites

**Figure 1:**
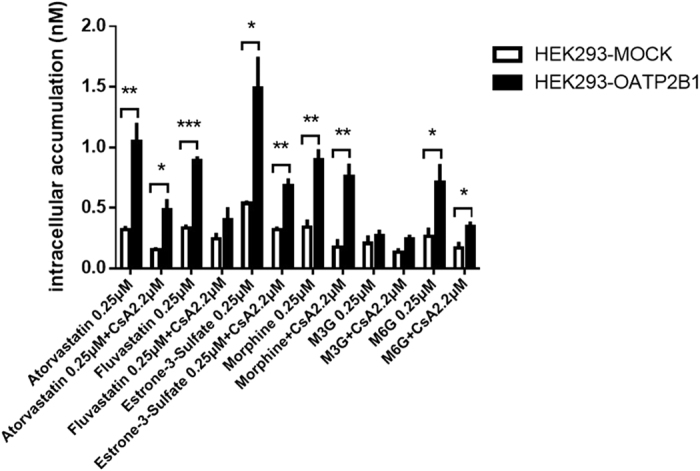
Intracellular accumulations for morphine, M3G and M6G determined by HPLC-MS/MS in HEK293-hOATP2B1 and HEK293-MOCK cells. Each final concentration of 0.25 μM for atorvastatin, fluvastatin, estrone-3-sulfate (E-3-S), morphine, M3G and M6G was incubated with HBSS (pH7.4) solution at 37 °C for 20 min. Cyclosporine A (CsA, 2.2 μM) was used as OATP2B1 inhibitor to co-incubate with all these compounds in HBSS (pH7.4) solution at 37 °C for 20 min. The data was expressed as mean ± SEM (n = 3). Pairwise comparisons were calculated by student t-test to calculate P values (*P < 0.05, **P < 0.01, ***P < 0.001).

To investigate whether morphine and its two main metabolites, M3G and M6G, are the substrates of OATP2B1, HEK293 cells transfected with the plasmids of hOATP2B1 (HEK293-hOATP2B1) and mock-vehicle (HEK293-MOCK) were established. OATP2B1 substrates including atorvastatin, fluvastatin and E-3-S were used as positive controls to test the cell functions. Cyclosporine A (CsA) as OATP2B1 non-specific inhibitor was applied to double check the cell functions with reported incubating concentration of 2.2 μM (IC_50_ in HEK293 cells)^25^. From the results, there were 2 to 3 fold increases of OATP2B1 activity measured in the HEK293-hOATP2B1 cells compared to HEK293-MOCK for each classical substrate including atorvastatin, fluvastatin and E-3-S (Fig. 1). Meanwhile, CsA can also effectively inhibit the intracellular accumulations for these substrates. It indicated that HEK293-hOATP2B1 cells have potent uptake activity and can be used in substrate uptake experiments. Then we found more than 2-time increases of intracellular accumulations of morphine and M6G in HEK293-hOATP2B1 cells compared to HEK293-MOCK (0.90 nM *vs.* 0.34 nM and 0.71 nM *vs.* 0.26 nM for morphine and M6G, respectively). However, M3G concentrations didn’t show any differences.

### Uptake kinetics and affinity analysis for morphine and M6G in OATP2B1 stable transfection cells

**Figure 2:**
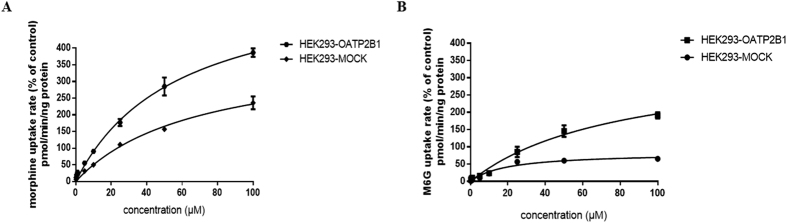
Uptake kinetic parameters evaluation for morphine and M6G. HEK293-hOATP2B1 and HEK293-MOCK cells were incubated (**A**) morphine and (**B**) M6G in HBSS (pH7.4) solution with final concentrations of 0.25 to 100 μM at 37 °C for 20 min. The data were expressed as mean ± SEM (n = 3).

The morphine and M6G affinity to OATP2B1 then was further explored by uptake assay. In the HEK293-hOATP2B1 cells, the Michaelis–Menten constant (*K*_*m*_) and maximum uptake velocity (*V*_*max*_) values of morphine and M6G were determined as 57.58 ± 8.90, 80.31 ± 21.75 μM and 608.9 ± 46.69, 353.1 ± 53.11 pmol/min/ng protein, respectively (Fig. 2A,B). Relative to morphine, M6G had a weaker affinity to OATP2B1 with 1.4-time higher *K*_*m*_ value than that of morphine.

### Effects of inhibitor and incubation pH on intracellular uptake of morphine and M6G in OATP2B1 stable transfection cells

**Figure 3:**
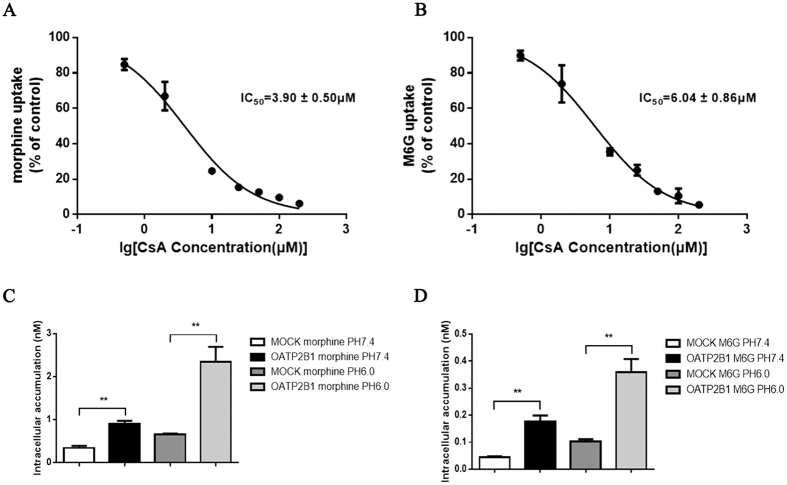
Effects of inhibitory and extracellular pH on morphine and M6G. The HEK293-hOATP2B1 and HEK293-MOCK cells were co-incubated with 0.25 μM (**A**) morphine or (**B**) M6G for 20 min at 37 °C with CsA (0.5 to 200 μM); 0.25 μM (**C**) morphine or (**D**) M6G were dissolved in pH7.4 or pH6.0 HBSS solution. After 20 min incubation at 37 °C. Each of intracellular concentration was determined by HPLC-MS/MS in HEK293-OATP2B1 and HEK293-MOCK cells. The data were expressed as mean ± SEM (n = 6). Pairwise comparisons were calculated by student t-test to calculate P values (**P < 0.01).

To further evaluate CsA inhibition of OATP2B1-mediated intracellular uptake of morphine and M6G. Its half-maximal inhibitory concentration (IC_50_) for morphine and M6G uptake was determined in HEK293-hOATP2B1 cells at room temperature, pH7.4. The results reflected that intracellular concentrations of morphine and M6G experienced a rapid decrease, and their IC_50_ values inhibited by CsA were 3.90 ± 0.50 μM and 6.04 ± 0.86 μM, respectively (Fig. 3A,B). In addition, morphine and M6G’s concentrations in HEK293-hOATP2B1 cells at acidic condition of pH6.0 were 2.62 fold and 2.05 fold, respectively, more than those at pH7.4 (Fig. 3C,D).

### RNAi mediated OATP2B1 knockdown in mouse brain endothelium (bEnd.3) cells

**Figure 4:**
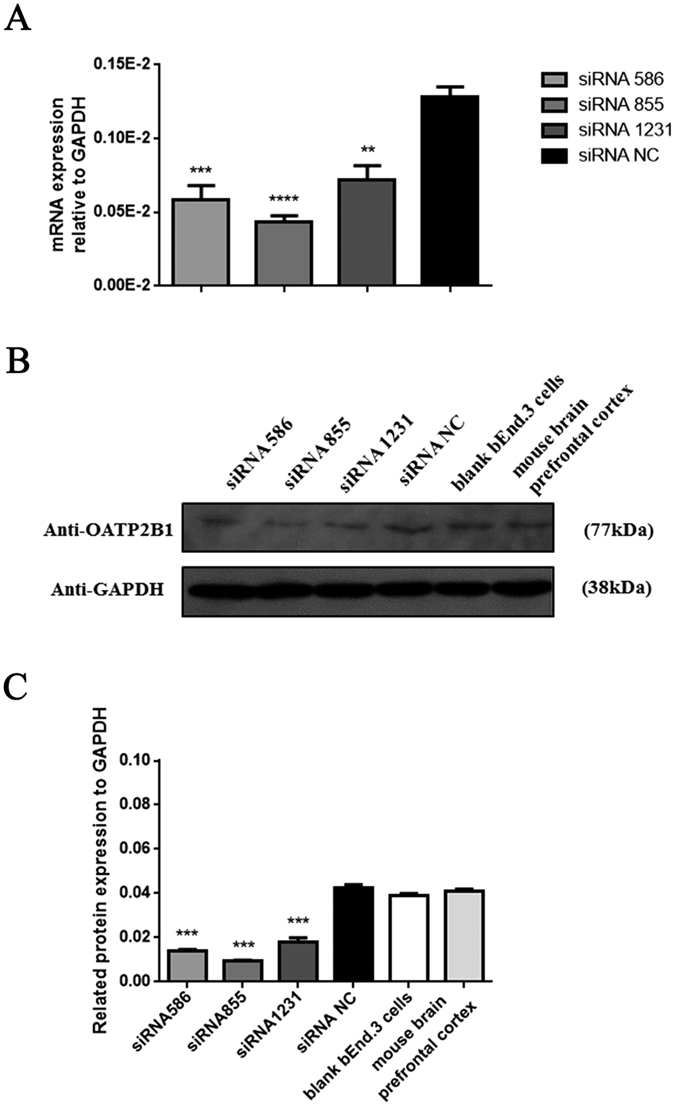
Knock down OATP2B1 in bEnd.3 cells by RNAi. (**A**) OATP2B1 mRNA expression detected by qPCR via three siRNA transfection. (**B**) OATP2B1 protein expression detected by Western blotting. (**C**) The average optical density (IOD) values for each protein stripe in the gel were normalized to GAPDH measured by Image Pro Plus 6.0 Software. All experiments were repeated three times under the same condition for counting. Pairwise comparisons were calculated by student t-test to calculate P values (**P < 0.01, ***P < 0.001, ****P < 0.0001) (n = 3).

We hypothesized that OATP2B1, as the transporter expressed in the brains, can mediate morphine and M6G crossing BBB *in vivo*. To confirm that, we expected to specially knock down OATP2B1 in mice brains. Firstly, three pairs of siRNAs (OATP2B1) (No. 586, 855 and 1231) were designed and validated in mouse brain endothelial cells, bEnd.3. From the result, siRNA-586, siRNA-855 and siRNA-1231 transfected bEnd.3 cells for 48 h can block OATP2B1. Especially, siRNA-855 owned a highest inhibition effect among them (Fig. 4A). Normalized to the average optical density values (IOD) of GAPDH, siRNAs (No. 586, 855 and 1231) downregulated OATP2B1 expression by 57.29%, 77.65% and 67.66%, respectively, compared to negative control (NC) siRNA group (Fig. 4B,C). Therefore, siRNA-855 was used to formulate with DGL-PEG/dermorphin for further studies since it had the highest knockdown efficiency.

### Preparation and function inspection of siRNA (OATP2B1) capsulated NPs

**Figure 5:**
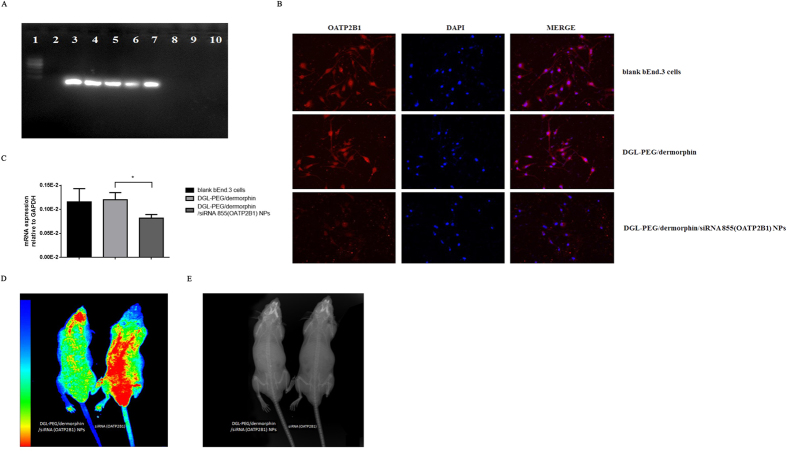
Investigate the character and stability of DGL-PEG/dermophin/siRNA (OATP2B1) NPs *in vitro* and *in vivo*. (**A**) The stability test for NPs was performed by formaldehyde modified gel electrophoresis with different weight ratios of 0.5:1, 1:1, 2:1, 4:1, 6:1, 10:1, 15:1, 20:1 for DGL-PEG/dermorphin to siRNA (OATP2B1); (**B**) The functions estimation of NPs were detected by immunofluorescence assay in bEnd.3 cells. Intensity of red fluorescence signal was revealing the target expression level; (**C**) mRNA expression of OATP2B1 was measured in bENd.3 cells after siRNA (OATP2B1) capsulated NPs transfected for 48 h, DGL-PEG/dermorphin and blank bEnd.3 cells are being as negative control. (**D**) DGL-PEG/dermorphin/siRNA (OATP2B1) NPs or siRNA transfected in nude mice for 1 h after anesthesia observed by *in vivo* imaging. (**E**) The X-ray imaging of nude mice reflected the actual tissues position. Pairwise comparisons were calculated by student t-test to calculate P values (*P < 0.05) in qPCR assay (n = 3).

DGL-PEG/dermorphin was prepared at a ratio of 3:1 (v/v) and diluted with 50 mM Na_2_SO_4_ DEPC solution. To obtain permanent NPs, material of DGL-PEG/dermorphin was capsulated siRNA(OATP2B1) at the ratios of 0.5:1, 1:1, 2:1, 4:1, 6:1, 10:1, 15:1 and 20:1 (w/w, to siRNA), then separated by formaldehyde gel electrophoresis. We found that the best ratio of DGL-PEG/dermorphin to siRNA is 10:1 (w/w). Under such circumstances, there were no clear stripes on the gel which indicated that siRNA capsulated NPs were completely stable under the electrophoretic migration (Fig. 5A). Immunofluorescence and qPCR assay showed that DGL-PEG/dermorphin didn’t impact the OATP2B1 in bEnd.3 cells, but siRNA capsulated NPs can significantly and specifically restrict it (Fig. 5B,C).

### Functions analysis of siRNA(OATP2B1) capsulated NPs *in vivo*

Nude mouse was injected with 156 μl solution of DGL-PEG/dermorphin/siRNA (OATP2B1) NPs (10:1, w/w; 3:1, v/v) from caudal vein. The siRNA (OATP2B1) capsulated NPs was labeled with cy3 dye (absorption wavelength was set at 525 nm; excitation wavelength was set at 565 nm). The same volume solution of siRNA (OATP2B1)-cy3 without DGL-PEG/dermorphin was injected to another nude mice as positive control. After mice received anesthesia for 1 h, the cy3 (red fluorescence) labelled siRNA distributed in both of mice bodies. Fluorescence signals were then tracked. Majority of NPs were delivered into the mice brains visualized by red fluorescence signals (Fig. 5D). X-ray images of mice bodies were used as controls (Fig. 5E). The result from Fig. 5E showed that siRNA without DGL-PEG/dermorphin delivery was distributed widely and randomly in different mice tissues.

### siRNA(OATP2B1) capsulated NPs mediated brain OATP2B1 downregulation and acute morphine tolerance reverse in mice

**Figure 6:**
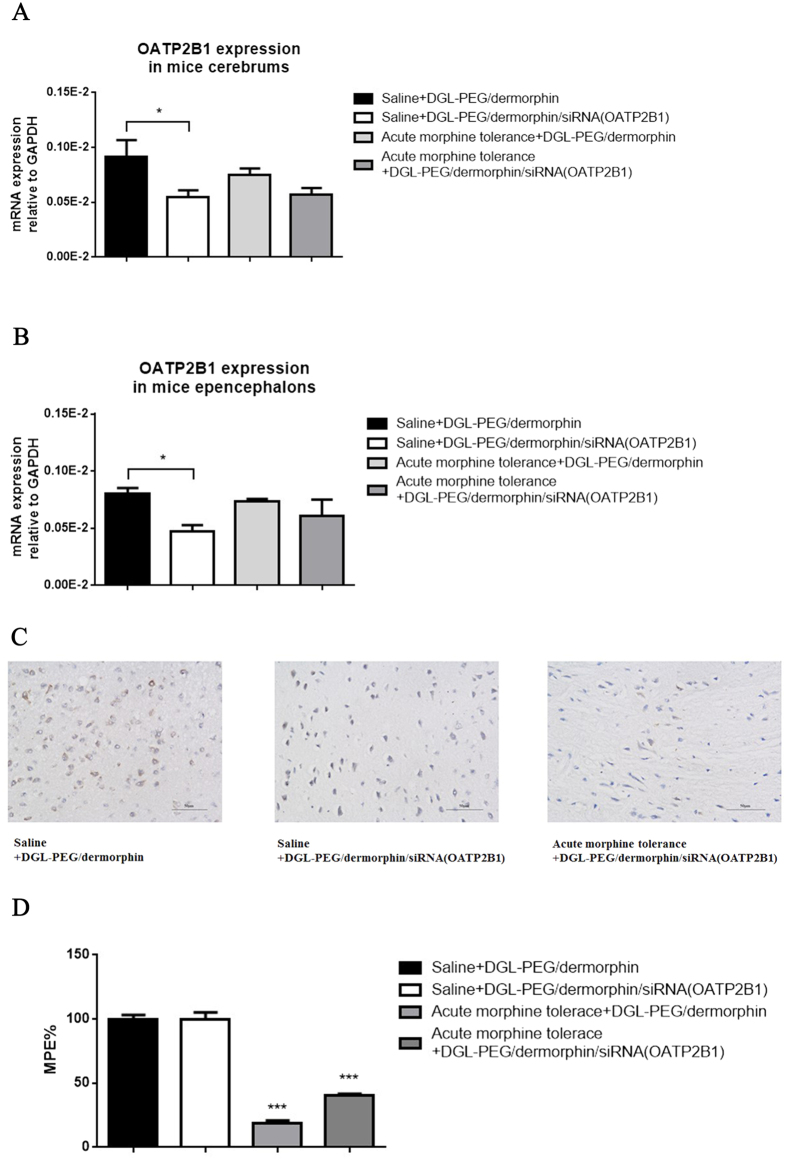
siRNA (OATP2B1) capsulated NPs function performance in acute morphine tolerance mice. OATP2B1 expression analyzed in mice (**A**) cerebrums or (**B**) epencephalons which treated with DGL-PEG/dermorphin or DGL-PEG/dermorphin/siRNA (OATP2B1) NPs Na_2_SO_4_ DEPC solutions for 48 h before morphine induced acute tolerance generates; (**C**) OATP2B1 (brown) detected by immunohistochemistry in brain coronal section of mouse; (**D**) The maximal possible effect (MPE%) was calculated from the data of trail-flick assay in each group of saline or morphine treated mice after acquiring with DGL-PEG/dermorphin or DGL-PEG/dermorphin/siRNA (OATP2B1) NPs Na_2_SO_4_ DEPC solutions for 48 h. Pairwise comparisons were calculated by student t-test to calculate P values (*P < 0.05, ***P < 0.001) (n = 6).

To further investigate the effects of NPs, each of 156 μl DGL-PEG/dermorphin diluted with 50 nM Na_2_SO_4_ DEPC solution was injected into one group of ICR mice as negative control, and the same volume of siRNA (OATP2B1) capsulated NPs (10:1, w/w; 3:1, v/v) solution was given to another group of mice (intrathecal injection, i.t.). After 48 h, the mice were treated with morphine (100 mg/kg, subcutaneous injection, s.c.) or the same volume of saline. A test dose of morphine (10 mg/kg, s.c.) was administrated at 4.5 h to verify the status of acute morphine tolerance. Then, mice cerebrums and epencephalons were harvested and used for qPCR and immunohistochemistry assay. We found OATP2B1 was decreased in normal or acute morphine tolerance mice cerebrums and epencephalons after siRNA(OATP2B1) capsulated NPs were injected (Fig. 6A,B). Similar trends like that less labeled areas (color brown) of OATP2B1 in normal or acute morphine tolerance mice brains can be observed after siRNA(OATP2B1) capsulated NPs were delivered (Fig. 6C). Compared to saline administrated mice, MPE% values were decreased by 81.3% and 59.8% in mice of acute morphine tolerance pretreated with DGL-PEG/dermorphin and siRNA(OATP2B1) capsulated NPs, respectively (Fig. 6D).

### Determination of morphine and its metabolites in mice cerebrums, epencephalons and plasma after brain OATP2B1 inhibition

**Figure 7:**
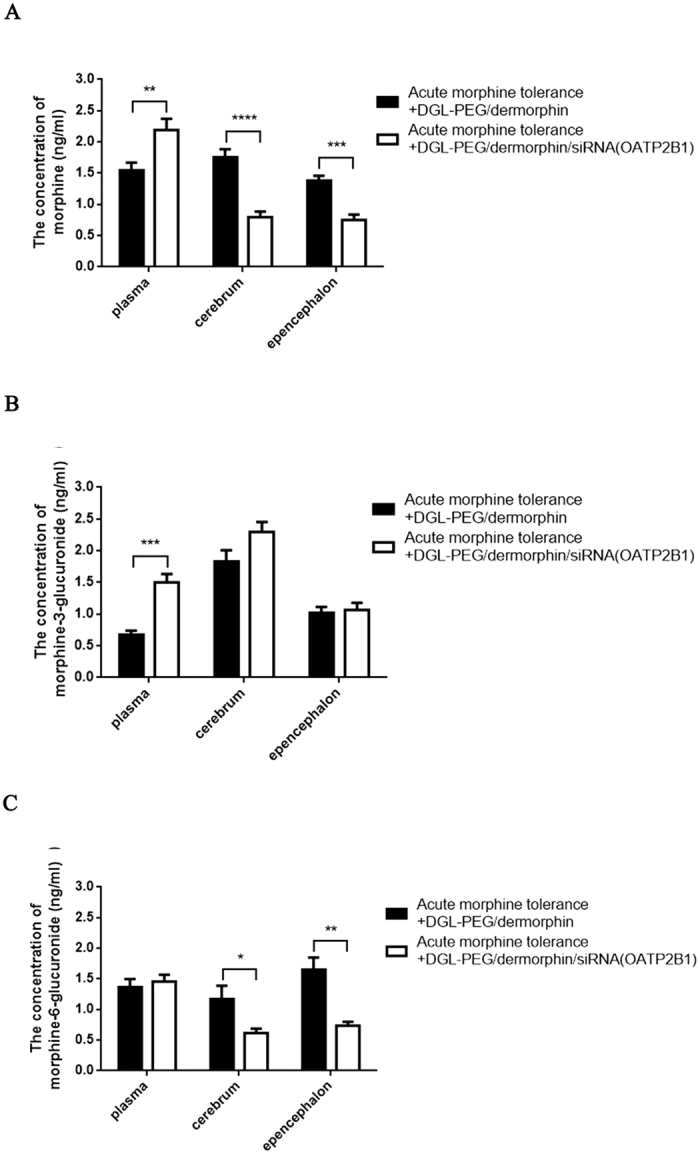
Concentrations of morphine, M3G and M6G in brain OATP2B1 inhibition mediated acute morphine tolerance mice brain-tissues or plasma. Concentrations of (**A**) morphine, (**B**) M3G and (**C**) M6G were determined by SPE-HPLC-MS/MS in acute morphine tolerance mice cerebrums, epencephalons and plasma with or without OATP2B1 knockdown in the brains. Pairwise comparisons were calculated using student t-test (*P < 0.05, **P < 0.01, ***P < 0.001, ****P < 0.0001) (n = 6).

To investigate whether OATP2B1 knockdown can impact the concentrations of morphine and its metabolites in mice cerebrums, epencephalons and plasma. Acute morphine tolerance mice were treated with siRNA (OATP2B1) capsulated NPs and DGL-PEG/dermorphin Na_2_SO_4_ DEPC solutions. From the results, the concentrations of morphine and M6G were significantly reduced in mice cerebrums and epencephalons, but not in the plasma (Fig. 7A,C). Although there were only slightly changes for the concentration of M3G in mice cerebrums and epencephalons, a noticeable increase for M3G in mice plasma can never be overlooked (Fig. 7B). To exhibit data in detail, the ratios of morphine to M3G and M6G to M3G were listed in Table 1.

### Regulation of proteins correlated with tolerance after brain OATP2B1 depression in mice prefrontal cortexes

**Figure 8:**
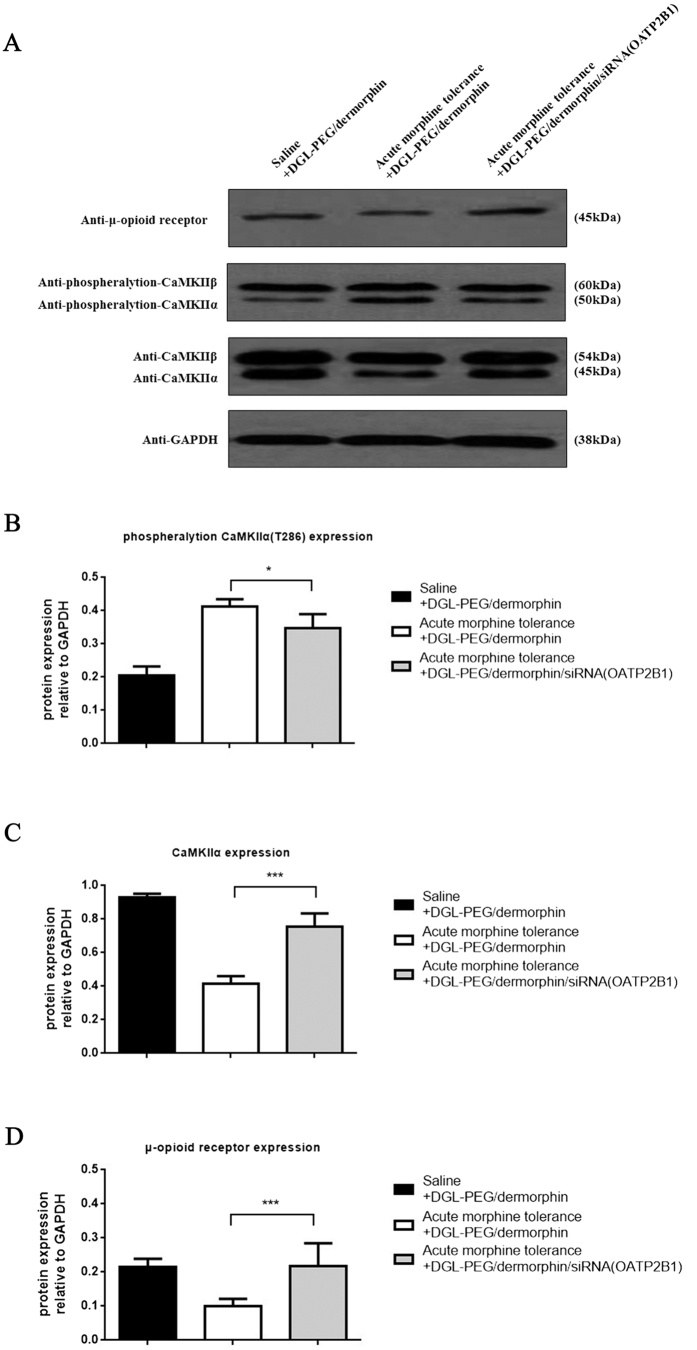
Tolerance correlated proteins expression in mPFCs after brain OATP2B1 inhibition. (**A**) phosphorylation of CaMKIIα, CaMKIIα and MOR protein expression in tolerance induced mPFCs with or without brain OATP2B1 knockdown. (**B**) phosphorylation of CaMKIIα at Thr286, (**C**) CaMKIIα, (**D**) MOR protein expressions in mPFCs normalized to average intensity optical density (IOD) values of GAPDH and measured by Image Pro Plus 6.0 software. All experiments were repeated three times under the same condition for counting. Pairwise comparisons were calculated by student t-test (**P < 0.01, ***P < 0.001) (n = 3).

To further demonstrate whether OATP2B1 knockdown in acute tolerance mouse brain will contribute to the expression changes of proteins correlated with tolerance. OATP2B1 was knocked down in normal or acute morphine tolerance mice brain by siRNA (OATP2B1) capsulated NPs. We extracted all the mice prefrontal cortexes (mPFCs) to further research. Based on the western blotting result, protein expression of MOR and CaMKIIα were all upregulated in morphine tolerance and reduced after OATP2B1 blocked (Fig. 8A,C,D). The phosphorylation of CaMKIIα at Thr286 showed a reverse trend under such conditions (Fig. 8A,B).

## Discussion

Our study used novel brain targeting NPs to deliver siRNA into mice brains. By this means, we investigated OATP2B1 transporter involved in morphine and M6G BBB transport. In addition, knocking down OATP2B1 in mice brains may contribute to a reverse of acute morphine tolerance.

Based on the results of uptake assay, it has been confirmed that morphine showed higher intracellular accumulations and stronger affinity to OATP2B1 than M6G. Meanwhile, in acidic conditions (pH6.0), morphine concentrations in HEK293-hOATP2B1 stable transfection cells are significantly higher than M6G. Trace the causes, we found that acidic extracellular pH would change the extent of the substrate binding pocket of the transporter^25^. Like OATPs, peptide transporter 1 (PEPT1) and mono-carboxylate transporter 1 (MCT1) are all exhibiting a pH sensitive activity. Lowering the extracellular pH would lead to an enhancement of uptake for their substrates^30^,^31^,^32^,^33^. Acidic extracellular pH can also increase the *V*_*max*_ of substrates of OATP2B1^34^. Since phenolic OH group dependent pKa of morphine is 9.26, lower than that of M6G which is 9.42 due to glucuronide hydrolysis, so morphine could be easier to dissociate under acid condition at pH 6.0 than M6G and lead to an increase of morphine intracellular accumulation through OATP2B1-mediated uptake^35^.

OATP2B1 mediates morphine and M6G uptake, but not M3G. *In vitro*, we didn’t see any change of intracellular accumulations of M3G in HEK293-hOATP2B1 cells. *In vivo*, inactive M3G experiences a slightly increase in the plasma of mice, but not significant in mice cerebrums or epencephalons. These results indicated that OATP2B1 should not be responsible for M3G BBB uptake. We found that the lipophilicity order for morphine and two metabolites at pH7.4 is morphine >M6G >M3G^35^. OATP2B1 are inclined to selectively transport the substrate which exhibits high lipophilicity. For example, atorvastatin owns a lower logP value than simvastatin^36^, but a relative higher affinity to OATP2B1, simvastatin is even hindered in OATP2B1-mediate intracellular uptake^37^.

We measured each concentration of morphine, M3G and M6G in mice plasma, cerebrums and epencephalons, then counted the concentration ratios of morphine to M3G and M6G to M3G. From the results, both of the ratios were all declined after siRNA capsulated NPs delivered into the mice brains of morphine induced acute tolerance. In the mice cerebrums and epencephalons, morphine and M6G were all barricaded to cross BBB and dissociated into milieu interne after brain OATP2B1 was inhibited. However, in the mice plasma, M3G increased and leaded to both ratios still remaining downtrends. That means M3G accumulations are more than morphine and M6G in the mice plasma. To explain this phenomenon, we realized some organic anion transporters can dimerize with organic cation transporters in the cell membranes and form chimeras. For example, in rats, organic anion transporter 1 (OAT1) can dimerize with organic anion transporter 1 (OCT1). Inhibiting rOAT1, the MPP^+^ intracellular uptake can also be cut down in rOCT1 and rOAT1 oligomerization stable transfection cells^38^. OATP2B1 downregulation may suppress other dimerized transporters in the BBB contributing to M3G uptake decrease or efflux increase. Compared to the integral brain uptake concentrations of M3G, these variations may not become a significant factor to impact its brain distribution.

When OATP2B1 was blocked, morphine and M6G were significantly barricaded to cross BBB, that may change the expression of the proteins correlated with tolerance in mPFC which is considering as a main neural regulation domain in the brain for tolerance observation^39^. In our study, MOR significantly recovered from internalized status in acute morphine tolerance, the same thing happened to CaMKIIα after siRNA (OATP2B1) came into play. Since MOR is a potential substrate of CaMKIIα^39^, both of them will experience same expression trends after tolerance developed. Some reports also referred this point. For example, MOR desensitization will be enhanced when constitutive activations of CaMKIIα in Xenopus oocytes^40^,^41^. On the other hand, contrasting to CaMKIIα, phosphorylation of it has a noteworthy decrease. Calcium influx induced by morphine tolerance may involve in this event. It can activate N-methyl-D-aspartate (NMDA) receptor and lead to CaMKIIα auto-phosphorylation at Thr286^42^. Both of NMDA receptor and CaMKIIα would exist positive feed-forward loops of phosphorylation in morphine tolerance^43^,^44^.

In summary, OATP2B1 can mediate morphine and M6G transport crossing BBB. The siRNA (OATP2B1) capsulated DGL-PEG/dermorphin NPs specifically restrict OATP2B1 expression in the mice. The morphine induced acute tolerance is suppressed through altering MOR and CaMKIIα expression or phosphorylation. These results may provide the basis for morphine antinociception enhancement.

## Materials and Methods

### Materials

Penicillin, streptomycin, fetal bovine serum (FBS), trypsin, Dulbecco’s modified Eagle medium (DMEM) were purchased from GIBCO (Invitrogen Life Technologies, USA). OATP2B1 rabbit polyclonal antibody was purchased from Biorbyt Company (Biorbyrt LLC, San Francisco, USA). Rabbit polyclonal antibody against MOR, CaMKII and phosphorylated CaMKII were purchased from the Abcam Company (Shanghai, China). Mouse monoclonal antibody against glyceraldehyde phosphate dehydrogenase (GAPDH) was purchased from KangChen Bio-tech Inc. (Shanghai, China). HRP-conjugated goat anti-rabbit IgG was purchased from MultiSciences Biotech Co, Ltd. (Hangzhou, China). The enhanced chemiluminescence (ECL) detection system was purchased from LumiGLO. BCA protein assay kit, immunofluorescence stationary liquid, DAPI staining fluid were obtained from Beyotime Institude of Biotechnology (Haimen, China). Alexa Fluor 488 Goat Anti-Rabbit IgG (H + L) Antibody, highly cross adsorbed was purchased from Invitrogen Life Technologies Company (San Jose, CA, USA). Keygen membranes extraction kit (KenGEN BioTECH, JiangSu, China).

Atorvastatin, fluvastatin, CsA and E-3-S (estrone-3-sulfate sodium salt) were purchased from the Sigma Aldrich Company (St. Louis, MO, USA). Morphine, M3G and M6G were purchased from Cerilliant Corporation (Round Rock, TX, USA). DGL-PEG/dermorphin materials were prepared by Prof. Jiang Chen in Fudan University (Shanghai, China). The siRNA (OATP2B1) (No. 586,866,1221), siRNA negative control labeled with cy3 dye and diethylpyrocarbonate (DEPC) were obtained from Sangon Biotech Company (Shanghai, China).

HPLC-grade methanol and formic acid were purchased from Tedia Company (Fairfield, OH, USA). Ultrapure water (18.2 MΩ) was obtained from an ELGA–pure lab Ultra system (High Wycombe, UK). M6G-d3 was labeled by Biomag System Company (Changshu, Jiangsu, China). LEICA inverted fluorescence microscope equipped with a 40× Plan pro lens.

### Cell culture

HEK293-hOATP2B1 and HEK293-MOCK cells which transfected with human OATP2B1 expression plasmid and control plasmid (mock-vehicle) to HEK293 cells (derived from human embryonic kidneys) were generously donated from Dr. Dafang Zhong in Shanghai Institute of Materia Medica, Chinese Academy of Sciences. The bEnd.3, a mice brain endothelial cell line, was kindly gifted by Dr. Fuqiang Hu in Institute of Pharmaceutics, Zhejiang University. All the cells were cultured at an atmosphere of 5% CO_2_ and 95% air at 37 °C and using Dulbecco’s Modified Eagle’s Medium (DMEM) supplemented with 10% fetal bovine serum (FBS), penicillin, streptomycin and trypsin.

### Animals

ICR mice (male, body weight of 20–25 g) and nude mice (male, body weight of 18–20 g) were obtained from Experimental Animals Department of Zhejiang University.

### Ethics

All the animal experiments were strictly conducted in accordance with the protocols approved by the Ethics Committee for Animal Studies at Zhejiang University. The paperwork was according to the documentation of ‘The Detailed Rules and Regulations of Medical Animal Experiments Administration and Implementation’ (Document No. 1998–55, Ministry of Public Health, China).

### Acute morphine tolerance and antinociception test for mice

The ICR mice of acute tolerance morphine were induced lasting for 6 h by the treatment of a high dose of morphine saline solution (100 mg/kg s.c.)^45^,^46^. An equal volume of saline was applied as control. To estimate tolerance, mice received a test dose of morphine (10 mg/kg s.c.). And the antinociceptive effect was determined at 4.5 h after morphine injection^47^,^48^. The model of acute morphine tolerance mice was validated by measuring the significant reduction of antinociceptive effect.

Tail-flick assay was performed to assess basal nociception and morphine induced antinociception as the previous report^46^,^47^. In brief, the distal one-third of mice tails was immersed into a 52 °C water bath and the latency to tail-flick response was simultaneously recorded. Morphine-induced antinociception was determined at 30 min after an injection of morphine (10 mg/kg s.c.) and it reflected as the percentage of maximal possible effect (MPE) based on the following formula:


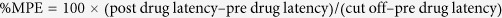


A cutoff time of 12 s was used to barricade mice tissue damage.

### Cellar uptake assay

HEK293-hOATP2B1 and HEK293-mock cells were seeded in poly-d-lysine-coated 24-well plates (Costar Corning Inc, USA) at a destiny of 2 × 10^5^/well. The uptake assay was performed after two days. To specifically eliminating the process, cells which experienced rigorously counting in each well were washed twice with PBS (pH7.4), then each drug was pre-incubated with 200 μl Hank’s balanced salt solution (HBSS, pH7.4) containing morphine, M3G, and M6G, respectively. Control group included OATP2B1’s substrates (atorvastatin, fluvastatin, E-3-S). CsA was used as classical inhibitor of OATP2B1. Each substrate or inhibitor was mixed with morphine, M3G and M6G respectively. Each of the final concentration of atorvastatin, fluvastatin and E-3-S was 0.25 μM in HBSS (pH7.4), while the CsA was 2.2 μM in HEK293 cells based on the reported IC_50_ value^25^. The pre-incubation was performed at 37 °C for 20 min. Then, cells were washed twice with ice-cold PBS (pH7.4) and lysed with 100 μl 0.1% SDS (sodium dodecyl sulfonate water solution). The amount of substrates in the cell lysates was analyzed by HPLC-MS/MS and normalized to each of the protein concentration measured by BCA assay.

To estimate the affinity and uptake kinetics parameters of OAPT2B1 substrates, the incubation concentration of both morphine and M6G was set at 0.25, 0.5, 1, 5, 10, 25, 50 and 100 μM.

For the assay of CsA effect on morphine and M6G uptake inhibition, the final incubation concentrations of morphine and M6G were 250 nM mixed with 0.5 to 200 μM CsA HBSS (pH7.4) solution respectively. For pH dependent studies, 250 nM morphine and M6G were mixed in HBSS (pH7.4) or HBSS (pH6.0). Acetonitrile (160 μl with internal standard M6G-d3) was added into 80 μl of the cell lysates to precipitate the proteins. Then, all the samples were centrifuged at 12,000 × g for 15 min. The supernatants were evaporated and dried in a centrifugal thickener. The residues were dissolved in 100 μl mobile phase (methanol: 0.05% formic acid (2:98, *v:v*) by vortex. An aliquot of 10 μl sample was injected into HPLC-MS/MS and the concentrations of compounds were determined.

### Tissues samples preparation using SPE

All the methods were referring to Yang *et al*.^49^. In brief, the precise dissection of each mouse cerebrum and epencephalon was performed, then 0.05% formic acid in purified water was added into them at 100 μl/mg tissue. The tissue homogenates were prepared at 200 mg/ml by using animal’s tissues auto-grind instrument (Tissueslyser-48, Shanghai Lixin Tech.) at a grind rate of 60 Hz for 2 min. Acetonitrile at a ratio of 2:1of solvent to homogenate was added to precipitate the protein in the samples. After centrifuging at 13,000 g for 10 min, the supernatant was separated. M6G-d3 (IS) was added into the supernatant with a final concentration of 10 ng/ml in each sample. Then all the mixture solutions were loaded onto balanced HLB Waters SPE columns activated by 5 ml methanol and 1 ml acetonitrile to elute. The collective liquid was evaporated and dried in a centrifugal thickener for HPLC-MS/MS determination.

### HPLC-MS/MS determination

All the samples including cell lysates, mice plasma and tissues were determined and quantified by HPLC-MS/MS. For high performance liquid chromatography, Agilent 1290 infinity LC system was equipped with a G4220A quaternary pump, G4226A auto sampler, and G1330B 1290 thermostat. For mass spectrum, AB SCIEX 4000 plus triple quadrupole mass spectrometer (AB SCIEX Technologies) was combined with an electrospray ionization source. The auto-sampler was maintained at 4 °C, and the temperature for column compartment was set at 30 °C. Chromatographic separations were achieved on an Agilent HILC PLUS SB-C18 column (2.1 mm × 50 mm, 3.5 μm). The mobile phase I for analyzing morphine, M3G, M6G, M6G-d3 (ISTD) consisted of 0.05% formic acid in purified water (A) and methanol (B) with a gradient elution of 95% A at 0–1 min, 98% B at 1–3 min, 98% B at 3–5 min and 5–6 min, 95% A at 6–7 min with a flowing rate of 0.25 ml/min. The mobile phase II for analyzing atorvastatin, fluvastatin, E-3-S, rosuvastatin (ISTD) consisted of 0.10% formic acid in purified water (A) and methanol (B) were followed with a gradient elution of 20% A at 0–1 min, 90% B at 1–3 min, 90% B at 3–4 min, 20% A at 4–5 min with a flowing rate of 0.30 ml/min.

The mass spectrometer with ESI source was operated in positive or negative ionization mode. The mass spectrometer parameters were set as following: collision energy, 36 eV for morphine and 43 eV for M3G, M6G, M6G-d3 (ISTD); 30 eV for atorvastatin, 40 eV for fluvastatin and rosuvastatin (ISTD), −45 eV for E-3-S; declustering potential, 89 V for morphine, and 85 V for metabolites and IS, 40 V for atorvastatin, 80 V for fluvastatin, −95 V for E-3-S. The integral temperature of drying gas, 350 °C; drying gas flow, 8 L/min; temperature for ionization 550 °C. Data were acquired using the Analyst 1.5.2 (AB SCIEX) in the multiple reaction monitoring (MRM) mode by recording ion currents for the following transitions: 286–200.9 m/z for morphine, 462.1–286.1 m/z for M3G and M6G, 465.1–289.1 m/z for M6G-d3 (ISTD), 536.2–415.4 m/z for atorvastatin, 412.3–224 m/z for fluvastatin, 482.3–258.1 m/z for rosuvastatin (ISTD) in positive mode. 349.2–269.2 m/z for E-3-S in negative mode.

### Synthesis and characterization of DGL-PEG/dermorphin

Combing reaction of DGL and α-Malemidyl-ω-N-hydroxysuccinimidyl polyethyleneglycol (NHS-PEG3500-MAL) based on a molar ratio of 1:5 (DGL: PEG) in PBS (pH 8.0) for 2 h at 37 °C. Surface amino groups of DGL were specifically united with NHS groups under the catalysis of PEG. DGL-PEGs, as final production, were purified by ultrafiltration through a membrane (cutoff × 5 kDa) in PBS (pH 7.0). Then dermorphin were recruited to react with purified DGL-PEGs at a molar ratio of 2:1 (dermorphin: DGL-PEG) in PBS (pH 7.0) for another 24 h at 37 °C. After that, DGL-PEG were specifically reacted with dermorphin, yielding the DGL-PEG-dermorphin polymer. The materials of DGL-PEG/dermorphin were experiencing freeze-dried, then dissolved in D_2_O and analyzed in a 400 MHz spectrometer (Varian, Palo Alto, CA, USA) to make an identification.

### Stability evaluation of NPs

The dermorphin-conjugated NPs were freshly prepared by mixing the DGL-PEG/dermorphin and siRNA (OATP2B1, No. 855) in 50 mM Na_2_SO_4_ DEPC solutions (3:1,v/v) with different weight ratios of 0.5:1, 1:1, 2:1, 4:1, 6:1, 10:1, 15:1, 20:1. The stability of the NPs were evaluated using 1% agarose gel electrophoresis by using TAE buffer, it was prepared with DEPC and mixed with formaldehyde and formamide and heated at 95 °C for 2 min prior to loading. The stability estimation of NPs was based on the gel-shift extent between siRNA and DGL-PEG/dermorphin. If the component can capsulate the siRNA, there is no band which can be observed after electrophoresis.

### siRNA transfection

The bEnd.3 cells were cultured in 6 well-plate, and transfected at high confluence (>70%). Three candidates of siRNA against mouse OATP2B1 including NC siRNA are siRNA-586 (SenseGCUGCUAGCUGCUUUCAAUTT, antisenseAUUGAAAGCAGCUAGCAGCTT), siRNA-855 (SenseCCAGUCACACAGAAACCAATT, antisenseUUGGUUUCUGUGUGACUGGTT), siRNA-1231 (SenseGGAGAAACAUGAGUUUCAUTT, antisense AUGAAACUCAUGUUUCUCCTT). NC siRNA (Sense UUCUCCGAACGUGUCACGUTT, antisense ACGUGACACGUUCGGAGAATT). All the siRNAs were transfected with Lipofectamine 3000 (Thermo Fisher Scientific, USA) into bEnd.3 cells. DGL-PEG/dermorphin and DGL-PEG/dermorphin capsulated siRNA (OATP2B1, No. 855) NPs were added into the bEnd.3 cells based on the ratios of 3:1 (v/v) and 10:1 (w/w). After cultivating 48 h, total RNA and proteins were extracted for the detection of related gene expression.

### Immunohistochemistry

The ICR mice were divided into 3 groups. One was injected DGL-PEG/dermorphin Na_2_SO_4_ DEPC solutions, other two groups were injected with DGL-PEG/dermorphin/siRNA (OATP2B1) Na_2_SO_4_ DEPC solutions for 48 h. One of siRNA (OATP2B1) capsulated NPs administrated groups was treated with morphine (100 mg/kg, s.c.) to induce acute tolerance, another two groups were as control using same volume of saline. After performing the antinociception test for each group, the brains of mice were harvested. Then, the tissues were fixed in 4% paraformaldehyde for 12 h then kept in cold 30% sucrose and PBS solution overnight. Coronal sections (50 μm) obtained by die through the olfactory bulb of mice were cut by freezing microtome and exposed to 0.3% H_2_O_2_ (final concentration in PBS) for reducing the endogenous peroxidase. After that, they were blocked with 10% goat serum (Santa Cruz, Los Angeles, CA, USA) in PBS, and incubated with primary OATP2B1 rabbit polyclonal antibodies (diluted with 1:100) overnight at 4 °C before HRP-conjugated goat anti-rabbit polyclonal secondary antibody (1:2000) incubated for 1 h. All sections were treated with a peroxidase substrate solution, 3, 3′-diaminobenzidine tetra hydrochloride (DAB; Vector Laboratories) and hematoxylin (Sigma, Sigma-Aldrich, Shanghai, China) was used as counterstaining reagent. The prepared slides were observed with a 400× of magnification under an Olympus BX41 microscope to measure the OATP2B1 expression. The negative control group without primary antibody was carried out with the same procedure as described above.

### *In vivo* imaging

Nude mice were injected with the Cy3-labeled siRNA (OATP2B1) NPs via tail vein at a dose of 10 μg siRNA/mouse. Images were taken by CRI *in vivo* imaging system (Maestro, USA) immediately and 1 h after anesthetization with 10% chloral hydrate.

### Quantitative real-time PCR

Total RNAs were isolated from the selected clones by using RNA simple Total RNA Kit (Tiangen, China), cDNA was synthesized using PrimeScript RT reagent Kit (Perfect Real Time, Takara, Japan). All the samples were mixed with SYBR Premix Ex TaqTM (Tli RNaseH Plus, Takara, Japan) and measured by StepOne plus Real-Time PCR system. Expression of the target mRNAs was normalized to the housekeeping gene glyceraldehyde-3-phosphate dehydrogenase (GAPDH). The primer pairs for musOATP2B1 (SLCO2B1) is: 5′-TTCCACAACATCAAGTTCTTTGTCC-3′ as forward, 5′-GCTTTTCCACTGTGGAGATGGAG-3′ as reverse, size of the amplicon is 103 bp; primer set for musGAPDH is: 5′-GAGAAACCTGCCAAGTATGATGAC-3′ as forward, 5′-AGAGTGGGAGTTGCTGTTGAAG-3′ as reverse, size of amplicon is 129 bp.

### Western blot analysis

The total proteins were extracted from the cell lysates or mPFCs, then homogenized in RIPA lysis buffer with 1 × PMSF. The protein concentration of each sample was determined with BCA kit. Protein samples were subject to SDS-PAGE and transferred onto PVDF membranes. The blots were incubated in blocking solution containing 5% non-fat milk in TBST buffer (100 mM Tris-HCl, pH7.4, 150 mM NaCl and 0.1% Tween 20) for 1 h at room temperature prior to the overnight incubation with primary antibody at 4 °C. The antibodies used were OATP2B1 (1:500), MOR (1:500), CaMKIIα (1:5000) and phosphorylation of CaMKIIα (1:2000). After washing three times to remove the primary antibody, the HRP-conjugated goat anti-rabbit IgG (1:2000) was used as secondary antibody for detection by using ECL system. Target proteins were visualized by exposing the membranes to a Kodak film for 10 min in dark room. GAPDH was paralleled by using as an internal reference for control the expression level of OATP2B1, MOR, CaMKIIα and phosphorylated CaMKIIα. Each bands’ IOD values were calculated by using Image-Pro Plus 6.0 Software.

### Cell immunofluorescence

The bEnd.3 cells were cultured on glass slides of 6-wells and transfected with DGL-PEG/dermorphin capsulated siRNA (OATP2B1, No. 855) NPs as previously referred. All the cells were fixed by stationary liquid at 4 °C overnight, then the slides were washed three times with PBST (1% Triton X-100 mixed with PBS, pH7.4). After washing, the slides were blocked with blocking solution of 5% non-fat milk in TBST buffer (100 mM Tris-HCl, pH7.4, 150 mM NaCl and 0.1% Tween 20) for 1 h at room temperature and followed by incubation with OATP2B1 rabbit anti-human polyclonal antibody (1:100) for 4 °C overnight. Then thrice washings were carried out by using TBST during the period of Alexa Fluor 488 goat anti-rabbit IgG (H + L) antibody incubation. Finally, the target gene expression was examined under fluorescence microscope at 400× of magnification. Nuclei were visualized by staining the dye of DAPI.

### Data analysis

Statistics data are expressed as mean ± SEM derived from three paralleled independent studies. The half-maximal inhibitory concentration (IC_50_) values were calculated by sigmoidal curve fitting of the log_10_ inhibitor concentrations versus the uptake inhibition of morphine and M6G by CsA using GraphPad Prism 5.0 (GraphPad Software Inc., San Diego, USA). The *K*_*m*_ and *V*_*max*_ were evaluated using GraphPad Prism 5.0 by fitting the concentration dependent uptake data to the equations:


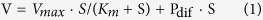


for HEK293-OATP2B1 cells.


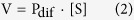


for HEK293-MOCK cells.

V is the initial uptake rate of the substrate (pmol/min/mg protein), S is the substrate concentration in the medium (μM) and P_dif_ is the uptake clearance corresponding to the passive diffusion and nonspecific binding to the cell surface. Unpaired two-side Student’s t-test was applied for comparisons of two groups’ data. A P value < 0.05 was considered statistically significant.

## Additional Information

**How to cite this article**: Yang, Z.-Z. *et al*. siRNA capsulated brain-targeted nanoparticles specifically knock down OATP2B1 in mice: a mechanism for acute morphine tolerance suppression. *Sci. Rep.* **6**, 33338; doi: 10.1038/srep33338 (2016).

## Figures and Tables

**Table 1:** The ratios of morphine to M3G and M6G to M3G were calculated after determined these compounds in acute morphine tolerance mice plasma, cerebrums and epencephalons with or without brain OATP2B1 inhibition.

Group	Tolerance + DGL-PEG/dermorphin		Tolerance + DGL-PEG/dermorphin/siRNA (OATP2B1)	
	[Morphine/M3G]	[M6G/M3G]	[Morphine/M3G]	[M6G/M3G]
Plasma	2.17 ± 0.59	2.01 ± 0.55	1.46 ± 0.23	0.97 ± 0.22
Cerebrum	0.89 ± 0.11	0.64 ± 0.17	0.34 ± 0.10	0.27 ± 0.07
Epencephalon	1.33 ± 0.45	1.62 ± 0.46	0.71 ± 0.10	0.60 ± 0.15

(Data are the mean ± SEM of 6 independent determinations).
